# Supplementary figures and images for: α-Synuclein plasma membrane localization correlates with cellular phosphatidylinositol polyphosphate levels
Source: eLife. 2021 Feb 15;10:e61951. doi: 10.7554/eLife.61951 (PMC7929559; doi:10.7554/eLife.61951)

siRNA-mediated gene silencing of  $\alpha$ Syn in A2780 cells

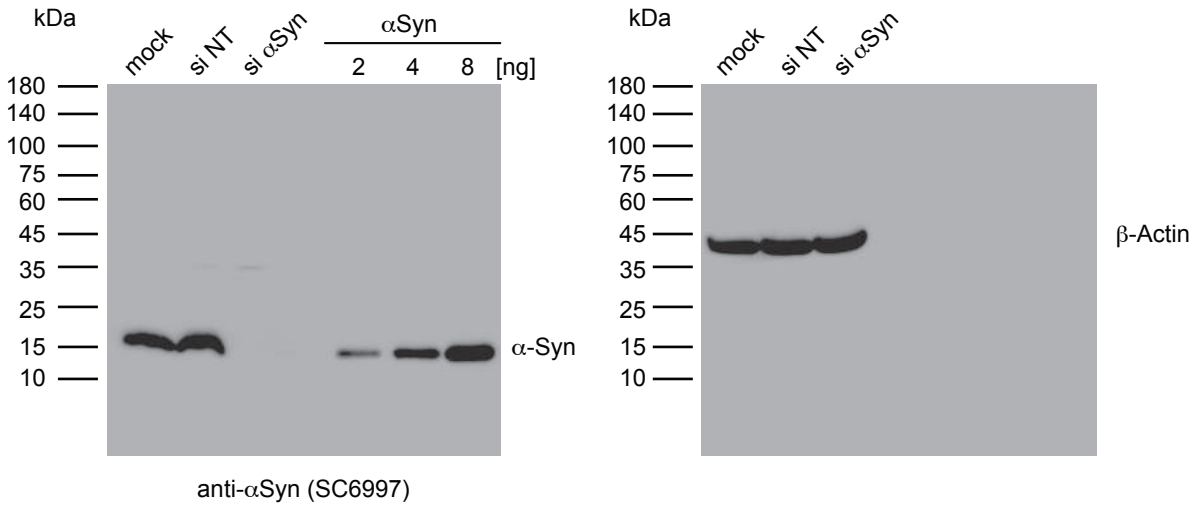

Supplement: Figure 1—figure supplement 1—source data 1. [file elife-61951-fig1-figsupp1-data1.pdf]

Endogenous  $\alpha$ Syn concentration increases during differentiation of SH-SY5Y cells

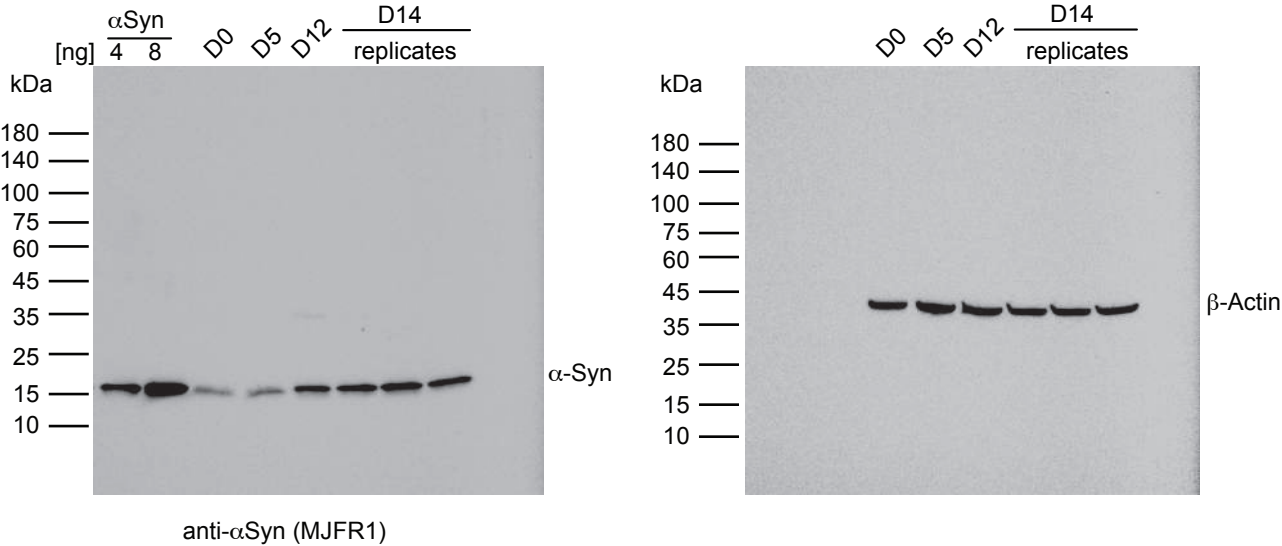

Supplement: Figure 1—figure supplement 1—source data 2. [file elife-61951-fig1-figsupp1-data2.pdf]

Endogenous  $\alpha$ Syn levels in commonly used human cell lines

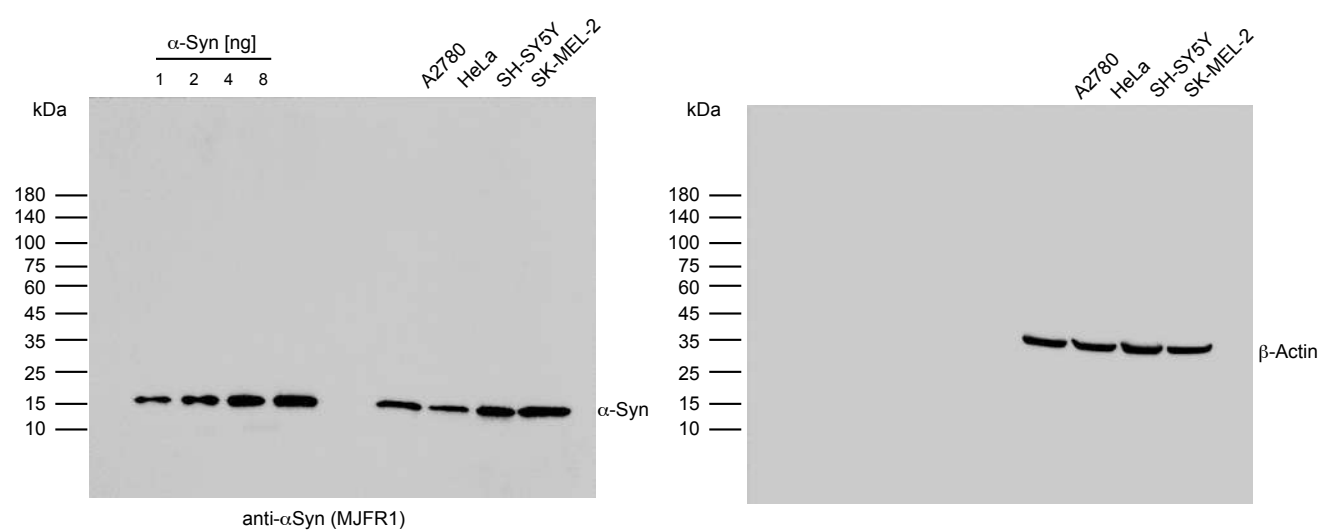

Supplement: Figure 1—figure supplement 2—source data 3. [file elife-61951-fig1-figsupp2-data3.pdf]

Endogenous insulin like growth factor receptor  $\beta$  (IGF-R $\beta$ ) in SK-MEL-2 and HEK 293 cells

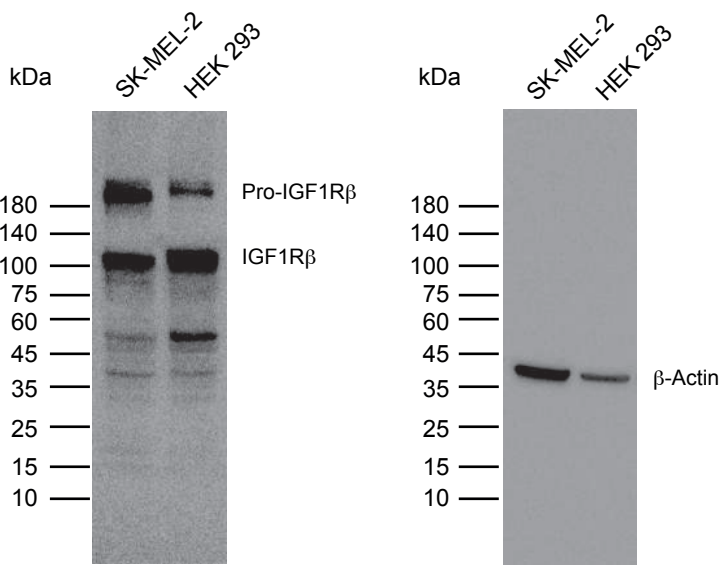

Supplement: Figure 3—figure supplement 1—source data 2. [file elife-61951-fig3-figsupp1-data2.pdf]
