# Supplementary material for: α-Synuclein plasma membrane localization correlates with cellular phosphatidylinositol polyphosphate levels
Source: eLife. 2021 Feb 15;10:e61951. doi: 10.7554/eLife.61951 (PMC7929559; doi:10.7554/eLife.61951)

Transient GFP, PIPKIγ (active or inactive) expression did not affect overall αSyn abundance in cells

A2780

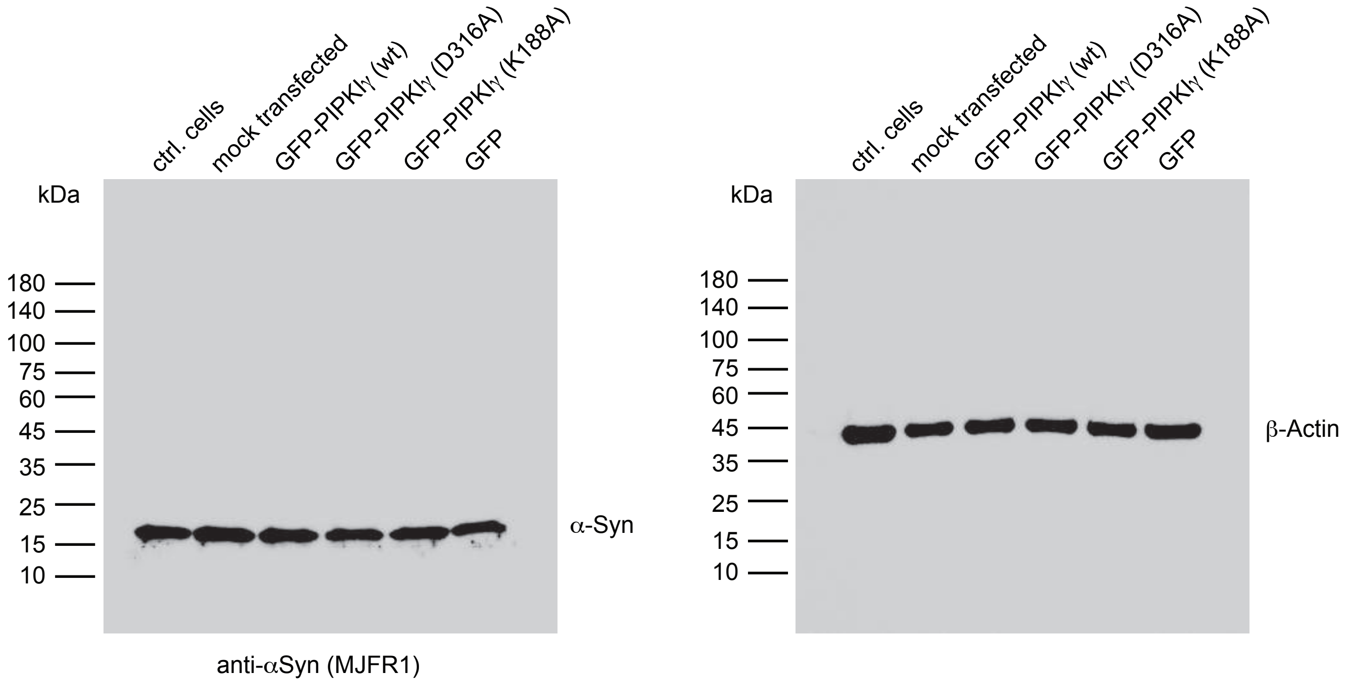

SH-SY5Y

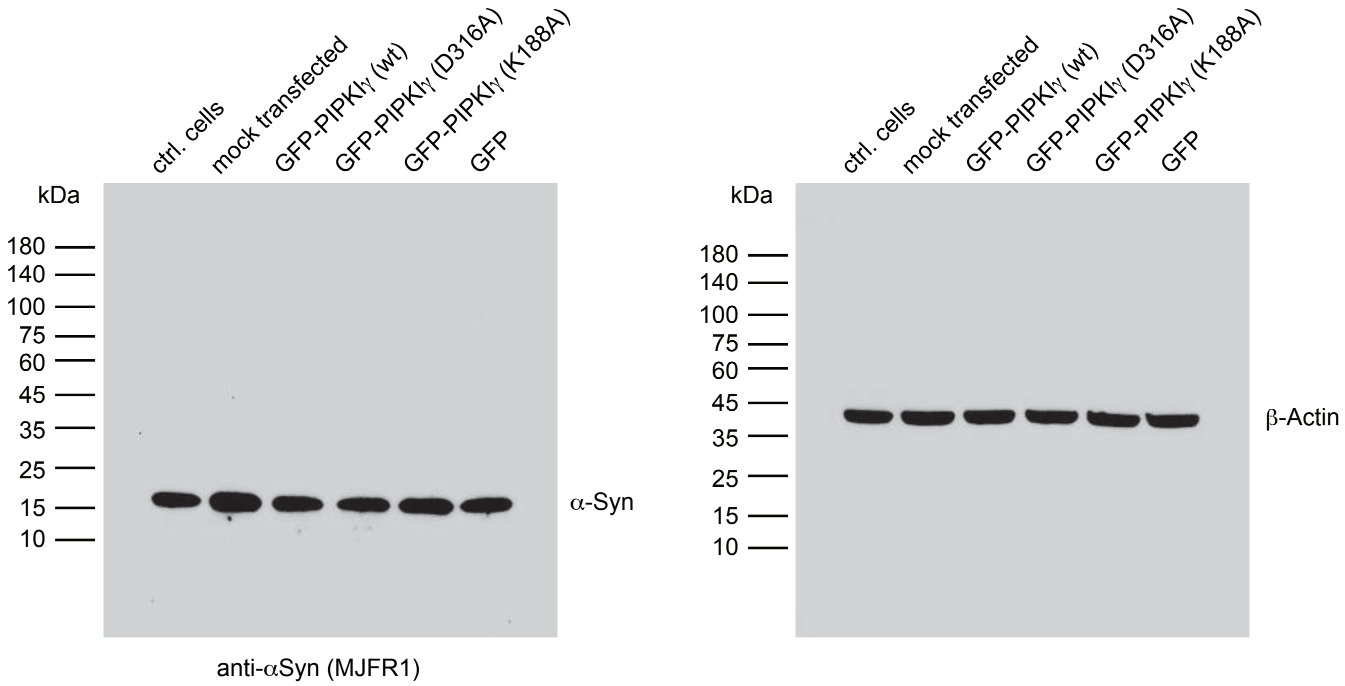

Supplement: Figure 1—figure supplement 2—source data 2. [file elife-61951-fig1-figsupp2-data2.pdf]
